# Towards an improved early diagnosis of neurodegenerative diseases: the emerging role of in vitro conversion assays for protein amyloids

**DOI:** 10.1186/s40478-020-00990-x

**Published:** 2020-07-25

**Authors:** Niccolò Candelise, Simone Baiardi, Alessia Franceschini, Marcello Rossi, Piero Parchi

**Affiliations:** 1 Department of Experimental, Diagnostic and Specialty Medicine (DIMES), University of Bologna, Bologna, Italy; 2 IRCCS, Istituto delle Scienze Neurologiche di Bologna, Ospedale Bellaria, Via Altura 1/8, 40139 Bologna, Italy; 3 Department of Biomedical and Neuromotor Sciences, University of Bologna, Bologna, Italy

**Keywords:** RT-QuIC, Biomarker, Diagnosis, Prion disease, Parkinson’s disease, Alzheimer’s disease, Lewy bodies, Prion protein, α-Synuclein, Tau

## Abstract

Tissue accumulation of abnormal aggregates of amyloidogenic proteins such as prion protein, α-synuclein, and tau represents the hallmark of most common neurodegenerative disorders and precedes the onset of symptoms by years. As a consequence, the sensitive and specific detection of abnormal forms of these proteins in patients’ accessible tissues or fluids as biomarkers may have a significant impact on the clinical diagnosis of these disorders. By exploiting seeded polymerization propagation mechanisms to obtain cell-free reactions that allow highly amplified detection of these amyloid proteins, novel emerging in vitro techniques, such as the real-time quaking-induced conversion assay (RT-QuIC) have paved the way towards this important goal. Given its high accuracy in identifying misfolded forms of prion protein from Creutzfeldt-Jakob disease (CJD) CSF, RT-QuIC has already been included in the diagnostic criteria for the clinical diagnosis of sporadic CJD, the most common human prion disease. By showing that this assay may also accurately discriminate between Lewy body disorders and other forms of parkinsonisms or dementias, more recent studies strongly suggested that CSF RT-QuIC can also be successfully applied to synucleinopathies. Finally, preliminary encouraging data also suggested that CSF RT-QuIC might also work for tau protein, and accurately distinguish between 3R- and 4R tauopathies, including Pick’s disease, progressive supranuclear palsy, and corticobasal degeneration. Here we will review the state of the art of cell-free aggregation assays, their current diagnostic value and putative limitations, and the future perspectives for their expanded use in clinical practice.

## Introduction

**Fig. 1:**
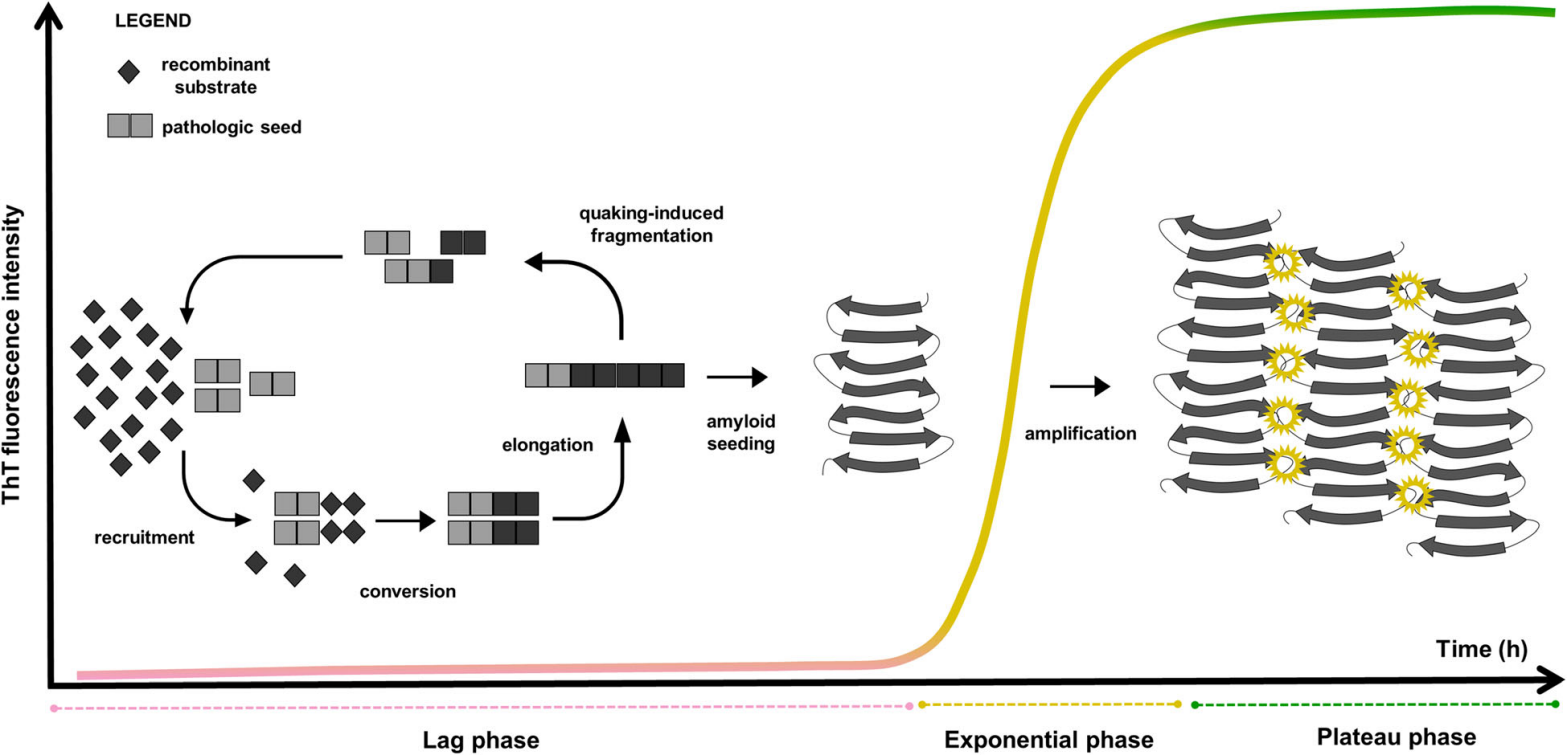
Schematic representation of the RT-QuIC reaction. The RT-QuIC reaction may be divided into temporal stages: the lag phase, the exponential phase and the plateau phase. The lag phase represents the time required for the reaction to take place. During this step, the seed is allowed to contact the substrate and to trigger the conformational change. Once enough energy is received by the system, ThT sensitive oligomers emerge, which incorporate the monomers into small aggregates (exponential phase). Eventually, when all the substrate is incorporated into fibrils, a plateau phase is observed

The most common neurodegenerative diseases feature protein misfolding, aggregation, and accumulation of protein amyloids inside or outside cells [17]. Consequently, the detection of these abnormal proteins in biofluids and accessible tissues represents the ideal candidate for the early antemortem identification of these disorders. However, owing to considerable differences in their concentration among various tissues and fluids, these protein aggregates are, in most cases, readily detectable by standard assays only in the affected brain. Thus, apart from Alzheimer’s disease (AD), in which changes in the CSF levels of certain tau and amyloid-beta isoforms reflect the ongoing deposition of these proteins in the brain, surrogate markers of neuronal damage such as 14-3-3, total tau and neurofilament proteins represented the only available biofluid markers to support the clinical diagnosis of neurodegenerative diseases until recently [85]. Although advancing ultra-sensitive immunoassays, enabling the detection of single molecules of the target of interest, allow the measurement of some of these markers also in the blood, making them a reliable tool for population screening, prognostic assessment, and monitoring of drug effect in clinical trials [3], the search for disease-specific markers remains the primary goal in the field. In this context, the recent development of ultrasensitive assays that indirectly reveal minute amounts of misfolded prion protein and other prion-like proteins, based on an amplification strategy, represented a major breakthrough in the field with enormous diagnostics potential for neurodegenerative diseases. In this work, we will review the state of the art of cell-free conversion assays and their current and foreseeable diagnostic applications in clinical practice. In particular, we will focus on the most promising of these assays, the real-time quaking-induced conversion (RT-QuIC), which uses a recombinant protein as substrate, intermittent shaking to foster conversion, and Thioflavin-T (ThT) to monitor the aggregation phase in real-time (Fig. 1) [91].

## Main text

### Search strategy and selection criteria

We searched Google Scholar and PubMed using the terms “prion”, “Creutzfeldt-Jakob disease”, “alfa-synuclein”, “tau protein”, “parkinsonism”, “Lewy body”, “tauopathy”, “Parkinson’s disease”, “MSA”, “PSP”, “CBD”, “AD” and “Pick’s disease”, each in combination with “real time-quaking-induced conversion” or “RT-QuIC” or “PMCA”. Studies and articles written in English were selected based on our rating of the methodological aspects and of their relevance to the diagnosis of human neurodegenerative diseases. A few articles providing reference to basic concepts or general information were added based on personal knowledge of the scientific literature.

### A brief history of in vitro conversion assays

The concept of cell-free aggregation assays stems from the discovery of the protein-only mechanism for the propagation of the prion protein (PrP) as the sole pathogenic agent of transmissible spongiform encephalopathies including CJD and other human and animal prion diseases, such as scrapie in sheep, and chronic wasting disease in cervids [68, 69]. Aggregation of PrP was found to be dependent on the secondary structure’s conformational changes, switching from α-helix enriched, as in the cellular prion protein, PrP^C^, into a β-sheet enriched conformation as in the abnormal scrapie prion protein, PrP^Sc^ [8, 15]. The modification of the secondary structure allows the misfolded protein to contact and convert its native counterpart, causing a positive feedback that eventually leads to aggregates and amyloid fibrils [70]. Moreover, biochemical features, such as protease resistance, detergent solubility, and thermal stability, are dramatically increased in the misfolded forms compared to the native protein [56, 77].

In the attempt to better understand PrP conversion, in the mid ‘90s, Kocisko and co-workers successfully generated aggregated species of PrP in a cell-free assay [47]. In this work, an excess of PrP^Sc^ isolated from prion-infected hamsters was partially denatured in guanidine-HCl and incubated with 35S-labelled recombinant PrP^C^ derived from uninfected hamster’s brain. The demonstrations of proteinase K (PK) resistance labeled PrP confirmed the de novo formation of misfolded species.

As a significant technical implementation to this assay, Saborio and co-workers [76] introduced cyclical sonication and incubation steps, which allowed to speed up the in vitro conversion and amplify as little as attograms of PrP^Sc^ in the presence of an excess of healthy brain homogenate as a source of PrP^C^. In this assay, termed protein misfolding cyclic amplification (PMCA), sonication provided the energy required to break aggregated species of PrP into misfolded, aggregation-competent species, while incubation allowed the conversion of the monomers into their pathogenic form. Through various sonication and incubation passages, the researchers were able to amplify the original PrP^Sc^ seeds greatly. As in the original assay, the detection relied on partial digestion with PK, followed by western blotting using specific PrP antibodies. Strikingly, PMCA was able to detect the presence of PrP^Sc^ in the brain of pre-symptomatic hamsters [86], rendering this method fundamental for both research and diagnostic applications. However, a significant limitation of the PMCA was that the end-products would still retain their infectiousness, posing a problem for the application in routine clinical practice. Moreover, PMCA appeared to be sensitive to contaminants, resulting in a suboptimal reproducibility [9, 20].

The use of bacterially expressed recombinant PrP as reaction substrate overcame the need for brain homogenates as the source of PrP^C^ [4], allowing the usage of a vast excess of the substrate facilitating the conversion into its pathogenic counterpart.

Methodological improvement of the aggregation assay included the swap of sonication with automated shaking, defined as quaking-induced conversion (QuIC). In addition to the clear advantages of an automated system, with this novel methodology, Atarashi and co-workers [4] were able to improve reproducibility compared to PMCA and shorten the time required to form further aggregates.

In parallel, Colby and co-workers [18] developed an assay for the detection of protein aggregates termed amyloid seeding assay, which relied on ThT for the detection of aggregate formation, thus bypassing the laborious and time-consuming detection by immunoblot. This fluorescent dye shows enhanced emission and a shift toward the red spectrum when bound to amyloid structures [10]. The scientists used purified mouse recombinant prion protein as the substrate for the seeded aggregation of either preformed fibrils derived from the same mouse PrP, brain homogenates from PrP infected mice, or CJD brain material, yielding a real-time rapid and ultra-sensitive (within attograms) detection of prions.

By combining the PMCA and QuIC cyclic amplification with the real-time detection of the products by ThT, Wilham and co-workers [91] developed the first version of the RT-QuIC, in which minute amounts of seed derived from brain homogenates were sufficient to trigger the aggregation of an excess of the recombinant prion protein, monitored in real-time by ThT (Fig. 1). The methodology was further improved to detect PrP seeds in the CSF [5], proving that the RT-QuIC could have a significant impact in the clinical practice.

From here, several studies tested the RT-QuIC for its diagnostic accuracy in prion disease and other neurodegenerative conditions. Nowadays, the diagnostic criteria for sporadic CJD (sCJD) already include RT-QuIC [94], and efforts are ongoing to expand the RT-QuIC application to the detection of other aggregation-prone proteins involved in the pathogenesis of neurodegenerative diseases.

**Fig. 2:**
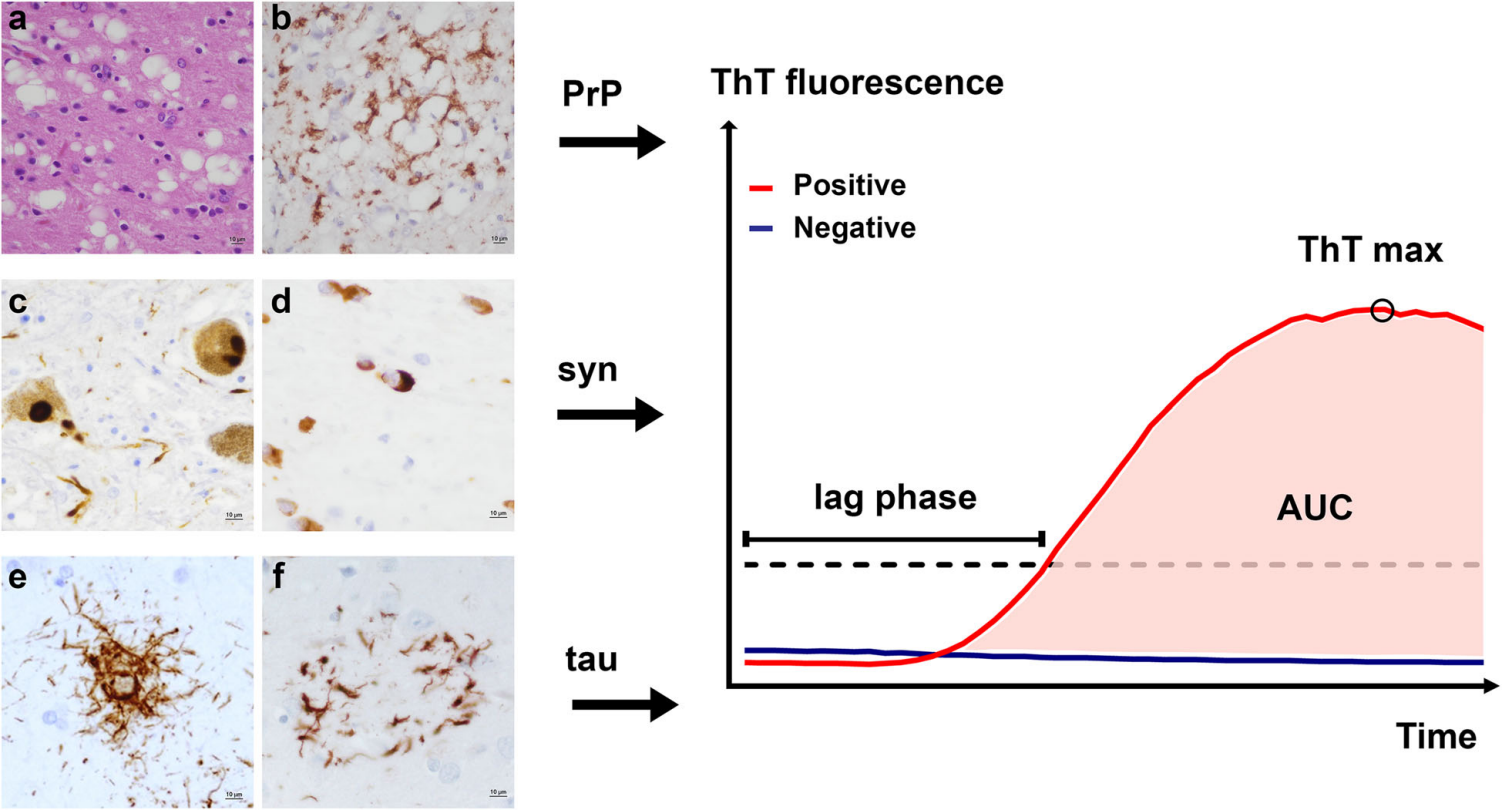
Main fields of application of the RT-QuIC assay and kinetic parameters analysed. Prion disease (**a**, **b**): spongiform change (**a**) and perivacuolar pattern of PrP^Sc^ deposition (**b**) in the occipital cortex of sCJD MM2C. Synucleinopathies (**c**, **d**): Lewy bodies and neurites in the substantia nigra of a DLB patient (**c**); glial cytoplasmic inclusions in the cerebellar with matter of a MSA case (**d**). 4R-tauopathies (**e**, **f**): tufted astrocyte (**e**), and astrocytic plaque (**f**) in the striatum of PSP and CBD cases. Hematoxylin and eosin stain (**a**), immunohistochemistry for PrP (mAb 3F4, dilution 1:400, Signet Labs, (**b)**, α-Syn (LB509, dilution 1:100, Thermo Fisher, (**c**, **d**), hyperphosphorylated tau (AT8, dilution 1:100, Innogenetics), (**e**, **f**). Lag phase is defined as the time interval between the beginning of the reaction and the time in which the curve of the fluorescent signal crosses the threshold (dot line); ThT max (circle) is defined as the maximum fluorescence value reached by the curve; AUC (area under the curve)

In the next sections, we will review the results with clinical diagnostic implications obtained to date with the three main RT-QuIC assays thus far developed, namely PrP RT-QuIC, alfa-synuclein (α-syn) RT-QuIC, and tau RT-QuIC (Fig. 2). A few studies on the so-called α-syn PMCA, performed with a protocol adapted to the same basic principles of the RT-QuIC, will also be reviewed.

### Diagnostic value of PrP RT-QuIC in rapidly progressive neurological syndromes

Despite their low prevalence, prion diseases encompass a broad spectrum of clinicopathological variants with significant heterogeneity in disease duration, symptomatology, and type or regional distribution of brain lesions. Current classification of prion diseases includes four major phenotypic entities, namely Creutzfeldt-Jakob disease (CJD), Gerstmann-Sträussler-Scheinker disease (GSS), fatal insomnia (FI), and variably protease-sensitive prionopathy (VPSPr) [7] (Table 1).

**Table 1:** Current classification of human prion diseases according to etiology and phenotypic features

Diseases	Etiology	Phenotype(s)^a^
Creutzfeldt-Jakob disease	Sporadic	MM(V)1 (typical/myoclonic variant) VV2 (ataxic/cerebellar or Brownell-Oppenheimer variant) MV2K (kuru-plaque variant) MM(V)2C (cortical variant) VV1 (cortico-striatal variant) Atypical MM1 with PrP-amyloid plaques in white matter
	Genetic	According to *PRNP* haplotype (mutation + codon 129) and PrP^Sc^ type
	Acquired	I. Iatrogenic (MM1, VV2, MV2K and MMiK^b^) II. Variant
Fatal Insomnia	Sporadic	MM2T (thalamic variant)
	Genetic	Fatal familial insomnia or MM(V)2T (thalamic variant)
Gerstmann-Sträussler-Scheinker disease	Genetic	According to *PRNP* haplotype (mutation + codon 129) and size/s of PrP^Sc^ fragment/s
Variably protease-sensitive prionopathy	Sporadic	According to *PRNP* codon 129 genotype and PK-resistance of PrP^Sc^ fragments

^a^The nomenclature and classification of sporadic Creutzfeldt-Jakob disease in distinct phenotypes is largely based on the combination of the genotype at codon 129 of *PRNP* (methionine, M, or valine, V) and the PrP^Sc^ type 1 or 2, as defined by the distinctive size (21 and 19 kDa) of their PK-resistant core

^b^i (=intermediate) refers to an electrophoretic mobility halfway between types 1 and 2; k = with PrP-amyloid kuru plaques

Moreover, some of these groups include disease subtypes with distinguishing molecular and clinicopathological features. Most significantly, CJD, by far the most prevalent prion disease in humans, comprises six subtypes, which are primarily determined by the genotype at the polymorphic codon 129 (encoding methionine, M or valine, V) in *PRNP*, and by the type (1 or 2) of abnormal prion protein (PrP^Sc^) accumulating in the brain (i.e., MM1, VV1, MM2, VV2, MV2) [63–65] (Table 1). The broad clinical heterogeneity at disease onset, which largely overlaps with that of other rapidly progressive dementias (RPDs), and the variable disease progression affecting survival times, significantly challenge the early diagnosis and clinical management of patients with prion disease [6]. Brain-derived CSF protein assays serving as surrogate markers for neuronal damage and diffusion-weighted magnetic resonance imaging (DW-MRI) have provided the primary support for the clinical diagnosis of sporadic prion disease in the past 15–20 years. However, both biomarkers demonstrated an incomplete diagnostic accuracy with significant variability across disease subtypes. For example, in sCJD, DW-MRI may disclose a typical, almost subtype-specific, lesion pattern in a subset of cases, including a high signal intensity in the basal ganglia and thalamus in the VV2 subtype, a prominent, often isolated, cortical signal hyperintensity in subjects MM2C or VV1 or marked asymmetry of the signal hyperintensity in the MM1 variant [94]. On the other hand, the same investigation may be unrevealing in 5–10% of cases, depending on the disease subtype and the time from disease onset, and yields a variable percentage of false-positive scans in RPDs with non-prion etiologies [90]. According to most studies, abnormal signal hyperintensities on fluid-attenuated inversion recovery (FLAIR) or DW-MRI involve 91–96% of sCJD cases and have an overall specificity of 87–98% [14, 75, 84, 90, 92, 93]. However, diagnostic accuracy was significantly higher in studies involving CJD surveillance centers than community hospitals [14]. In this regard, it is worth noting that CJD surveillance centers, likely as a consequence of improved technology and increased awareness of DW hyperintensities, have been witnessing an increase of referrals due to MRI findings of isolated cortical ribboning in patients with seizures, hyponatremia, Wernicke’s encephalopathy or even ischemia, often in the absence of typical clinical features of CJD. To limit the occurrence of such false-positive results and favor specificity, only hyperintensities involving the striatum (caudate and putamen nuclei) or at least two neocortical regions in different lobes except for the frontal (or both), are considered “typical” for CJD by current diagnostic criteria [93]. By applying these restrictive criteria to a large patient cohort, a recent study showed a 96.5% specificity but only a 73.2% sensitivity for CJD diagnosis [11]. Of notice, however, when more permissive criteria were applied (i.e., an abnormal signal in at least one region among the cerebral cortex of all lobes, caudate, putamen, and thalamus), the authors did not report any loss in specificity (95.5%) besides the expected increase in sensitiviy (92.7%) [11].

Similarly, CSF assays based on surrogate markers such as protein 14–3-3 and t-tau yield false-positive results in at least 10% of clinically selected cases (as for MRI, the erroneous use of these markers as a screening test in all RPDs increases this percentage significantly), with a wide diagnostic specificity range among studies (between 57 and 97%), Moreover, despite an overall good sensitivity (range 81–96%), they have relatively poor performance (sensitivity varies from ~ 70% in MV2K to ~ 10% in VPSPr) in atypical subtypes such as MV2K, MM2C, MM2T, and VPSPr [1, 19, 50]. Thus, clinical parameters and codon 129 genotype need to be taken into account with the diagnostic investigations’ results to achieve the most accurate clinical diagnosis [62, 93]. Finally, in the absence of a post-mortem neuropathological examination, an unknown number of atypical prion cases skip recognition.

The recent development of the RT-QuIC assay provided a reliable and robust tool to improve the early diagnosis of human prion diseases [94]. Results obtained in several laboratories with the so-called first generation of this assay (also termed previous QuIC-CSF, or PQ-CSF), which uses a full-length (23–231) hamster recombinant prion protein as substrate, demonstrated a 73–100% sensitivity and, most significantly, a 98–100% specificity in CJD (Table 2) [5, 13, 50, 54, 59, 81].

**Table 2:** CSF RT-QuIC protocols and diagnostic accuracy in prion disease

PrP Substrate	Reaction buffer	Parameters	Cohort size (N)^**a**^	Sens.	Spec.	Ref.
Hu 23–231	0.06–0.1 g/L substrate, 15 μL seed, 500 mM NaCl	30 s circular shaking, 30 s incubation, 2 min break, 37 °C	18 def sCJD	83%		[5]
			35 def non-CJD		100%	
			25 prob. sCJD	100%		
			130 ctrl		100%	
			16 prob. sCJD	87%		
			14 ctrl		100%	
	0.05 g/L substrate, 5 μL seed, 500 mM NaCl		24 gCJD	83%	-	[81]
			20 GSS	90%	-	
			12 FFI	83%	-	
Ha 23–231	0.1 g/L substrate, 15 μl seed, 300 mM NaCl	1 min double orbital shaking 600 rpm, 1 min rest, 42 °C	cohort 1: 56 def sCJD	91%		[54]
			52 ctrl		98%	
			cohort 2: 67 def sCJD	87%		
			52 ctrl		100%	
			15/13 def/prob. sCJD	79%		[59]
			43 ctrl		100%	
			179/97/29 def/prob./pos sCJD	81%		[50]
			46 gCJD	91%		
			348 ctrl		99%	
			24/26 def/prob. sCJD	73%	–	[13]
Ha 14–128 - Sheep 141–234 (chimera)	0.1 g/L substrate, 15 μL seed, 170 mM NaCl	1 min double orbital shaking, 600 rpm, 1 min rest, 42 °C	64 def sCJD	80%		[22]
			39 gCJD	100%		
			400 ctrl		99%	
Ha 90–231	0.1 g/L substrate, 15–30 μL seed, 300 mM NaCl, 0,002% SDS	1 min double orbital shaking 700 rpm, 1 min rest, 55 °C	10/1 def/prob. sCJD	91%	-	[60]
			43/5 def/prob. sCJD	96%		
			39 ctrl		100%	
			36/68 def/prob. sCJD	94%		[36]
			64 ctrl		100%	
			111 sCJD	92%		
			15 gCJD	93%		[30]
			67 ctrl		98.5%	
			63 sCJD, 14 ctrl^b^	95%	100%	
			10/12 def/prob. sCJD	95%		[13]
			17 ctrl		100%	
			116/73 def/prob. sCJD	93%		[31]
			33 gCJD	100%		
			100 ctrl		100%	
			61/41 def/prob. sCJD	96%		[29]
			80 ctrl		100%	
			439 def sCJD	93		[72]
			31 gCJD	97%		
			69 ctrl		98.5%	

*Abbreviations*: *Hu* Human, *Ha* Hamster, *prob* Probable, *pos* Possible, *def* Definite, *ctrl* Controls, *sens.* Sensitivity, *spec.* Specificity, *sCJD* Sporadic CJD, *gCJD* Genetic CJD, *FFI* Fatal familial insomnia, *GSS* Gerstmann-Sträussler-Scheinker disease

^a^Results obtained in disease groups with an n of 9 or lower have been omitted

^b^Prospective cohort

The assay demonstrated a good reproducibility in the inter-laboratory setting and across various CSF storage conditions, such as short-term CSF storage at different temperatures, long-term storage, repeated freezing and thawing cycles [21, 55]. The use of a chimeric version of PrP rather than wild-type hamster PrP yielded similar diagnostic accuracy in a population of both genetic and sporadic CJD, and a few FFI cases [22].

Modifications in the protocol, leading to the RT-QuIC of the second generation (also termed improved QuIC CSF, or IQ-CSF), allowed the experimental run time to be reduced to 1–2 days only and lead to a further overall increase in the sensitivity of the assay without affecting specificity [60]. These modifications included the increase in temperature during incubation, the addition of sodium dodecyl sulfate (SDS) to the reaction mix, and the substitution of full-length PrP with a truncated form of hamster PrP (90-231) as substrate. After the initial application of the IQ-CSF to a small cohort of samples, several groups successfully applied the new protocol to larger cohorts comprising in total about 1000 prion cases, comprehensive of the broad spectrum of prion disorders, and 500 controls [13, 29–31, 36, 72] (Table 2). Two independent studies specifically compared the first and second generation RT-QuIC [31, 36] in the same patient population, and both of them confirmed the higher sensitivity of the IQ-CSF compared to the first generation assay (Table 2).

Regarding the application of RT-QuIC to other tissues and body fluids, recent promising studies that used either the olfactory mucosa or skin biopsies as seed showed high test sensitivities from 89 to 100%, suggesting equal or even better diagnostic accuracy than CSF RT-QuIC [29, 51, 59, 61].

In summary, regardless of the type of protocol (Table 2), all studies conducted to date demonstrated the remarkable added diagnostic value of prion RT-QuIC compared to surrogate biomarkers. However, some methodological issues remain, limiting the full application of the assay as a screening test in RPDs. Indeed, RT-QuIC is a complicated and costly assay, which requires a continuous supply of recombinant protein, which is often challenging to recruit for laboratories that do not produce the substrate in-house. Moreover, the second generation RT-QuIC still needs validation in the inter-laboratory setting.

Finally, questions about the assay’s presumed absolute specificity and the effect of prion strains and disease subtypes on test sensitivity deserve further discussion.

### Current unsolved questions and potential limitations

#### Is prion RT-QuIC fully specific?

The development of an RT-QuIC assay with sufficient sensitivity requires a high concentration of recombinant substrate and a reaction environment favoring the seeded aggregation mechanism. Consequently, there is a need to monitor any spontaneous substrate aggregation that may generate false-positive signals. To this aim, samples are loaded four times in the standard RT-QuIC set-up, and a definite positive read requires that at least two of the four replicates generate a signal above a pre-set threshold, whereas a single positive well is considered an unclear result, prompting the repetition of the assay.

Some studies on prion RT-QuIC reported a few unexpected positive results [22, 30, 50, 54]. However, most of them involved patients who did not receive an autopsy confirmation, leaving open the possibility that they were clinically unrecognized atypical prion cases. Indeed, a retrospective analysis of such cases favors this interpretation. As an exception, Foutz and co-workers [30] reported a positive RT-QuIC in a patient with the neuropathological diagnosis of dementia with Lewy bodies (DLB). In order to investigate this sample deeply, the researchers precipitated the brain homogenate in phosphotungstic acid, a commonly used compound that allows the enrichment of pathogenic prions. Western blot analysis of the pellet revealed low but above-threshold levels of PK-resistant PrP^Sc^, which led to the conclusion that this might have been a case of subclinical prion disease, who died from DLB comorbidity. In other recent reports [38, 72], an autopsy-verified case of steroid-responsive encephalopathy with status epilepticus and one with dementia of mixed type (AD and vascular) also received a false-positive result, respectively, by PQ and IQ-CSF RT-QuIC. Thus, although false readouts by prion RT-QuIC seem to be extremely rare and some of them may even represent real prion cases, the test does not seem to provide yet a level of diagnostic certainty comparable to a neuropathological examination. Consequently, currently updated diagnostic criteria still require a post-mortem examination for the definite diagnosis of sporadic or acquired prion disease [94].

#### To what extent does the disease subtype affect the sensitivity of the assay?

Only a few studies examined a significant number of CJD stratified according to the disease subtype. Using the first generation of the assay, Cramm et al. [22] reported that neither the PrP^Sc^ type nor the codon 129 genotype alone had a significant impact on the RT-QuIC response. Nevertheless, PrP^Sc^ type 1 patients carrying MM at codon 129 showed a faster response that those carrying MV or VV (who most commonly harbor PrP^Sc^ type 2). Moreover, disease duration, which, once again, mainly depends on the disease subtype, had an impact on the test results, with patients with a shorter disease course (who most commonly have PrP^Sc^ type 1) showing higher seeding efficiency in the RT-QuIC response. In a large study including 186 pathologically confirmed sCJD cases with subtype classification, [50] RT-QuIC sensitivity varied according to the codon 129 genotype and was higher in MM (84.2%) than in MV (72.2%) or VV (79.5%) cases. Besides, there was convincing evidence that the assay sensitivity was significantly lower in the atypical subtypes linked to PrP^Sc^ type 2 (i.e., VV2, MV2K, MM2T, and MM2C).

As far as the second generation RT-QuIC is concerned, Foutz et al. [30] found that RT-QuIC positivity was significantly associated with older age, a shorter disease course, and a higher frequency of ataxia, features that are all linked to the two most common subtypes, MM1 and VV2. Furthermore, RT-QuIC was more frequently negative in sCJD MM2. However, there were no correlations between the RT-QuIC result and codon 129 polymorphism alone, gender, electroencephalography, or MRI findings. Franceschini et al., [31] in a large cohort of cases with disease subtype classification, found comparable levels of sensitivities in the three most common subtypes, namely MM(V)1, VV2 and MV2K, but confirmed the findings of reduced sensitivity of IQ CSF RT-QuIC in both MM2 subtypes (i.e., MM2C and MM2T or sFI). Similarly, Rhoads and co-workers recently reported that atypical sporadic prion disease subtypes (i.e., sFI, VPSPr, sCJD VV1, and sCJD MM2C) are more likely to have false negative RT-QuIC results [72].

As for the sCJD subtypes, only a few studies tested a significant number of cases affected by genetic prion disease. Using PQ RT-QuIC, Sano et al. [81] analyzed a small cohort of CSF specimens from patients with genetic CJD (E200K, *n* = 22; V203I, *n* = 2), GSS (P102L, *n* = 20) or FFI (D178N, *n* = 12). Results were comparable between the groups, with only a slightly higher sensitivity in GSS compared to FFI and gCJD (90% vs. 83.3%). In contrast, other groups, using different protocols, consistently reported a reduced sensitivity of RT-QuIC in FFI (0–57%), and GSS (0-50%) compared with gCJD (83.3–100%) [12, 22, 30, 31, 72]. Moreover, RT-QuIC gave positive results in one asymptomatic E200K carrier, who did not develop the disease after a 1-year follow-up [88]. Finally, based on the few results obtained in GSS and CJD cases carrying rare *PRNP* mutations, it is very likely that different disease-associated mutations might influence the test’s diagnostic value [31]. The outstanding performance of prion RT-QuIC, and the discovery that other proteins involved in neurodegenerative diseases show a seeding-conversion behavior similar to that of PrP, prompted the scientific community to develop an RT-QuIC assay that could detect protein amyloids in more common neurodegenerative diseases such as Alzheimer’s disease (AD) and Parkinson’s Disease (PD) in the CSF of diseased individuals.

In the next sections, we will review the successful adaptation of the RT-QuIC for the detection of pathogenic forms of α-syn and, to a lesser extent, of tau in human CSF.

### Diagnostic value of α-syn RT-QuIC in synucleinopathies

Synucleinopathies, including PD, DLB, and multiple system atrophy (MSA) are neurodegenerative diseases characterized by the intracellular accumulation of toxic α-syn aggregates [87] forming Lewy bodies (LB) and neurites in PD and DLB and oligodendroglial cytoplasmic bodies, and much rarer neuronal inclusions in MSA [24]. Similar to prion diseases, synucleinopathies manifest an extensive phenotypic heterogeneity involving clinical presentation, response to therapy, and rate of progression (Table 3). Moreover, the recognition that two other rarer syndromes, namely pure autonomic failure (PAF) and isolated rapid eye movement (REM) sleep behavior disorder (iRBD), characterized by the early α-syn accumulation in the peripheral autonomic system and lower brainstem, represent prodromal syndromes of both MSA and LB-related disorders (LBD), has widened the clinical spectrum of synucleinopathies and further complicated the early clinical diagnosis of these disorders [40, 46]. Increasing evidence demonstrating structural differences in the α-syn aggregates between MSA and LBD suggests that different conformational strains of α-syn may contribute to their clinicopathological heterogeneity [41]. Nowadays, even the early in vivo diagnosis of a full-blown LBD can be tricky since it requires the distinction from both atypical parkinsonisms such as progressive supranuclear palsy (PSP) and corticobasal degeneration (CBD), which are primary tauopathies, and other neurodegenerative dementias such as AD or frontotemporal dementia (FTD). Despite the progressive improvement of the accuracy of diagnostic criteria for the identification of synucleinopathies in vivo, the clinicopathological rate of accordance in autopsy verified cohorts is often less than optimal (i.e., 92.6% for PD, 81.6% for DLB, 62–78.8% for MSA) [48, 58, 67, 73].

**Table 3:** The spectrum of synucleinopathies: Main clinical features and diagnostic tests

Disease	Mean duration	Main affected brain regions	Main clinical features	Main diagnostic tests
PD	10–20	Brainstem	L-DOPA responsive parkinsonism	DaTSCAN (ioflupane)
DLB	8	Neocortices, limbic system, brainstem	Cognitive decline with fluctuations, parkinsonism, visual hallucinations, changes to cognitive ability, RBD	DaTSCAN (ioflupane), MIBG, PSG, EEG
MSA	7–9	Striatum, cerebellum, brainstem	Autonomic failure with various degree of parkinsonism, cerebellar and pyramidal signs	DaTSCAN (ioflupane), MRI, autonomic tests, FDG-PET
PAF	^a^	PNS	Isolate autonomic failure	Autonomic tests
iRBD	^a^	Lower brainstem	IRBD	PSG

*Abbreviations*: *PD* Parkinson’s disease, *DLB* Dementia with Lewy bodies, *MSA* Multiple system atrophy, *PAF* Pure autonomic failure, *iRBD* Isolate REM sleep behavior disorder, *PNS* Peripheral nervous system, *MIBG* Metaiodobenzylguanidine, *PSG* Polysomnography, *EEG* Electroencephalography, *FDG-PET* Fluorodeoxyglucose-positron emission tomography

^a^ Duration as “isolate” disorder, and rate of phenotypic conversion are highly variable

Moreover, current diagnostic criteria usually require the association of clinical findings, multiple diagnostic investigations, and an adequate follow-up of several years, to reach the accurate identification of the disorder [34, 66]. Most significantly, imaging, neurophysiological and neuropsychological tests are only supportive of the clinical findings, since they are not specific markers of the pathologic process (i.e., the accumulation of abnormal α-syn aggregates). Thus, in the era in which both clinical and scientific interests focus on the early recognition of neurodegenerative diseases, post-mortem neuropathology remains the gold standard for the definite diagnosis of synucleinopathies given the lack of an in vivo specific molecular marker.

Increasing evidence supporting the prion-like behavior of α-syn, together with those indicating that the seeding conversion mechanism could be replicated in vitro by aggregation assays such as PMCA [43, 82], prompted researchers to adapt the RT-QuIC assay for the detection of pathogenic α-syn. The key assay parameters and the main results obtained in the studies completed to date are shown in Table 4.

**Table 4:** CSF RT-QuIC protocols and diagnostic accuracy in synucleinopathies

Syn substrate	Reaction buffer	Parameters	Cohort size (N)	Sens.	Spec.	Ref.
WT Hu (commercial)	0,1 g/L substrate, 15 μL seed, 100 mmol/L PB	37 zirconia/silica bead, 1 min double orbital shaking 200 rpm, 14 min rest, 30 °C	cohort 1 (definite cases):			[27]
			12 pure DLB	92%		
			17 mixed DLB + AD	65%		
			13 AD+incidental LB	15%		
			20 ctrl, 30 AD		100%	
			cohort 2:			
			20 PD	95%		
			15 ctrl		100%	
			53 PD	84%		[89]
			17 MSA	35%		
			14 other synucleinopathies	86%		
			26 non α-syn parkinsonism		89%	
			52 ctrl		98%	
			10 PD	90%		[32]
			15 PD-LRRK2	40%		
			16 LRRK2 carriers	19%		
			10 ctrl		80%	
			105 PD	96%		[44]
			79 ctrl		82%	
			52 iRBD	90%		[42]a
			9 ctrl		78%	
K23Q Hu (home-made)	0,1 g/L substrate, 15 μL seed, 0,0015% SDS	6 silica beads (0.8 mm diameter), 1 min double orbital shaking 400 rpm, 1 min rest, 42 °C	17 DLB	94%		[37]
			12 PD	92%		
			16 AD		100%	
			12 ctrl		100%	
WT Hu (home-made)	0,1 g/L substrate, 15 μL seed, 100 mmol/L PB	37 zirconia/silica bead, 1 min double orbital shaking 200 rpm, 14 min rest, 30 °C	cohort 1 (definite cases):			[12]
			7/20 pure/mixed DLB, 1 MSA	93%		
			49 ctrl		96%	
			cohort 2:			
			20 DLB	85%		
			6 poss DLB	0%		
			10 AD		100%	
	0,1 g/L substrate, 15 μL seed, 0,0015% SDS	6 silica beads (0.8 mm diameter), 1 min double orbital shaking 400 rpm, 1 min rest, 42 °C	15 PD	100%		[52]
			11 ctrl		100%	
			cohort 1 (definite cases):			[74]
			21 LB-disorders	95%		
			101 ctrl		98%	
			cohort 2:			
			71 PD	94%		
			34 DLB	97%		
			28 PAF	93%		
			18 iRBD	100%		
			17 AD		84%	
			30 PSP/CBS		100%	
			31 MSA		93.5%	
			62 ctrl		98%	
	1 g/L substrate, 40 μL seed, 100 mM PIPES	1 min shaking 500 rpm, 29 min rest, 37 °C	76 PD	88.5%		[83]
			10 DLB	100%		
			10 MSA	80%		
			83 ctrl		94%	
			94 PD	94%		[82]
			75 MSA	85%		
			56 ctrl		100%	

Diagnostic groups/cohorts are meant to be clinically defined as probable cases, unless otherwise specified. Sensitivities values for diagnostic groups ≤5 are not included

*Abbreviations*: *Hu* Human, *WT* Wild type, *LB* Lewy bodies, *DLB* Dementia with LB, *AD* Alzheimer’s disease, *PD* Parkinson’s disease, *MSA* Multiple system atrophy, *iRBD* Isolate REM sleep behavior disorder, *PSP* Progressive supranuclear palsy, *CBS* Corticobasal syndrome, *PAF* Pure autonomic failure, *prob* Probable, *poss* Possible, *ctrl* Controls

^a^A preliminary not peer-reviewed draft of this study has been posted as preprint

The first successful application of α-syn RT-QuIC [27] involved a limited cohort of CSF derived from clinically diagnosed PD (*n* = 20) and neuropathologically confirmed DLB (*n* = 12) cases. Results showed a 92–95% sensitivity and a 100% specificity against 35 healthy controls and 5 PSP/CBD cases. Interestingly, three samples derived from patients affected by iRBD also gave a positive result, providing preliminary evidence that, in the clinical frame of a prodromic syndrome, the RT-QuIC may predict the future onset of a synucleinopathy. In an adaptation of the PMCA technique for the detection of misfolded α-syn that firmly reminds the RT-QuIC, as it makes use of a recombinant substrate, intermittent shaking and ThT, Shahnawaz et al. [83] measured α-syn seeding activity in PD patients (*n* = 76) and controls with other neurological diseases (*n* = 97). This novel α-syn PMCA assay distinguished PD from other neurologic disorders with 88.5% sensitivity and 97% specificity (Table 4). The research group also found that α-syn PMCA values in PD significantly correlated with disease severity according to the scores on the Hoehn and Yahr scale.

Whereas the original assay [27] showed an average lag phase of 50–60 h and required an observation time of 120 h, modifications of the experimental setup [37], including the use of a novel recombinant protein carrying the K23Q mutation, allowed the reduction of the lag phase to approximately 20 h and the total assay duration to 48 h. Most significantly, sensitivity remained above 90% and specificity 100% for both PD (*n* = 12) and DLB (*n* = 17) against AD cases (*n* = 16) and controls (*n* = 12).

To further explore its diagnostic value in the clinical contest, the authors applied α-syn RT-QuIC to CSF samples from a prospective observational cohort of 118 patients with parkinsonism of uncertain clinical diagnosis at lumbar puncture (LP) [89]. The overall diagnostic accuracy in distinguishing α-synucleinopathies from other disorders and controls was 84% (sensitivity = 75%, specificity = 94%), with a significant decrease in accuracy in comparison to the two previous studies, which analyzed relatively small groups of typical and, for the most part, neuropathologically confirmed LBD cases. Intriguingly, sensitivity significantly correlated with the type of synucleinopathy, being 100% in DLB, 84% in PD, and only 35% in MSA.

In another study, the RT-QuIC was used to detect α-syn seeding activity in patients carrying mutations in LRRK2 (Leucine-rich repeat kinase 2 gene) [32], the most common cause of genetic PD. In a blind analysis that compared these carriers (LRRK2-PD, *n* = 15; asymptomatic carriers, *n* = 16) with either idiopathic PD or healthy controls, the assay sensitivity was surprisingly rather low (40% in LRRK2-PD, 19% in the asymptomatic carriers) in the former group, whereas it was comparable to previous studies (90%) in the idiopathic (non-genetic) PD group. The assay specificity, however, dropped to 80%. The finding supports the prevalent idea that LRRK2 carriers may not necessarily have an underlying synucleinopathy. Indeed, neuropathological studies demonstrated, at least in a few of such cases, a nigral degeneration in the absence of protein deposition [53] or the presence of aggregates made up by misfolded tau [39].

The high sensitivity of α-syn RT-QuIC for the diagnosis of DLB was confirmed in another study evaluating a series of atypical cases referred to laboratories specialized in CJD diagnosis because of a rapidly progressive course [12]. The diagnostic accuracy, calculated on the cases assessed neuropathologically (*n* = 77), irrespectively of the clinical diagnosis (i.e., only 8 out of 27 LBD cases had a clinical diagnosis of probable DLB) resulted in a 92.8% sensitivity and 95.9% specificity. Additional results obtained in a clinical cohort of 36 cases showed an 85% sensitivity in the group of probable DLB and, intriguingly, a 0% sensitivity in those (*n* = 6) with possible DLB. Finally, there were no positive reads also in a group of 10 cases with a clinical diagnosis of probable AD.

In the first attempt to cross-validate α-syn seeding aggregation assays developed by independent laboratories, Kang et al. [44] undertook a blinded study in which the two participating groups (Soto’s lab and Green’s lab) analyzed CSF from the same subjects in the BioFIND cohort [45], including 105 clinically typical and well-defined moderate PD patients and 79 healthy controls. Despite the different denominations (RT-QuIC and PMCA), the two assays did not differ significantly in the basic principles. Overall, 92% of subjects showed concordant results across both assays. Eleven subjects were positive by RT-QuIC but negative by PMCA, and four subjects were positive by PMCA but negative by RT-QuIC. Sensitivity and specificity for the PMCA and RT-QuIC assays were 95.2%/89.9%, and 96.2%/82.3%, respectively. Clinical characteristics of false-positive and -negative subjects were not different from true-negative and -positive subjects, respectively.

In a recent study, using the standard protocol developed by Caughey’s lab, but a wild-type recombinant α-synuclein substrate rather than the mutant form used by Groveman et al. [37], Rossi and colleagues [74] applied the RT-QuIC to the largest cohort of CSF samples examined to date. They included 439 clinically well-characterized, or post-mortem verified, patients with parkinsonism or dementia, and, for the first time, a significant number of patients with iRBD and PAF. The RT-QuIC assay detected α-syn seeding activity across the spectrum of LBD, including DLB, PD, iRBD, and PAF, with an overall sensitivity of 95.3%. Surprisingly, all but two patients with MSA showed no α-syn seeding activity in the applied experimental setting. Finally, the assay demonstrated 98% specificity in a neuropathological cohort of 101 cases lacking LB pathology. In a recent study [82], a modified α-syn PMCA discriminated between PD and MSA pathologies based on the fluorescent signal profile in a cohort of 94 samples of CSF from patients with PD, 75 from patients with MSA and 56 from control individuals with other neurological diseases. The overall sensitivity for the identification of PD and MSA vs. controls was 93.6 and 84.6%, respectively, and, in both cases, specificity was 100% (Table 4). Furthermore, PD was correctly distinguished from MSA in 146 of the 153 samples analyzed, resulting in an overall sensitivity of 95.4%.

In the most recent of these studies, Iranzo and colleagues [42] applied the RT-QuIC developed by Fairfoul et al. [27] in 52 polysomnographically-confirmed iRBD patients, and only nine, matched, healthy controls. The assay gave a positive result in 47 (90.4%) patients and two (22.2%) controls. During follow-up, 32 (61.5%) patients received a diagnosis of PD or DLB 3.4 ± 2.6 years after LP, and 31 (96.9%) of these were positive for CSF α-syn RT-QuIC, while none of the controls developed a synucleinopathy.

Lastly, following the successful application of PrP RT-QuIC on peripheral tissues, De Luca et al., [23] applied the RT-QuIC to detect misfolded forms of α-syn in brushes of olfactory mucosa. However, in contrast to the high accuracy in prion disease, α-syn RT-QuIC showed a suboptimal performance (65.5% sensitivity, and 84.4% specificity). Notably, a few PSP samples gave a definite read, suggesting a certain degree of cross-reactivity with misfolded tau.

### Current limitations and future perspectives

The results of the studies mentioned above demonstrated that RT-QuIC is highly accurate in diagnosing synucleinopathies, with values almost as good as those previously obtained in prion disease. However, they also showed divergent findings and significant variability in the predictive values of the test, which are likely related to critical variables such as the choice of recombinant substrate and experimental setting, the accuracy of the final diagnosis (i.e., neuropathological vs. clinical), and the number of examined cases in each diagnostic group.

For example, specificity was very high among studies focusing on neuropathologically verified cohorts (95.9–100%) [12, 27, 37, 74], with two of them reporting a full-specificity [27, 37]. In these cohorts, false-positive readouts involved only four cases, two with AD, one with Wernicke’s encephalopathy, and one with unspecified encephalitis [12, 74]. However, whereas the two cases reported by Rossi et al. [74] showed a consistent positivity in repeated analyses, the other two [12] gave an opposite negative result when re-analyzed after breaking the blinded code. Thus, while the latter two cases suggest a true false-positivity due to the self-aggregation of the recombinant α-syn during the RT-QuIC reaction, the consistent positive signal in the former two samples is also compatible with a real α-syn seeding activity. Indeed, the presence of LB pathology could not be rule out in these cases, given that the histopathological examination did not include the olfactory bulb [2, 71], or the peripheral autonomic ganglia [16, 33], which can be first sites of α-syn accumulation.

In contrast to neuropathologically verified cohorts, the results obtained in clinical groups consistently showed false-positive reads [12, 32, 42, 44, 74, 89], resulting in a drop of specificity. The incomplete specificity was particularly evident with the assay developed by Green’s lab, which is also the most frequently applied to date. Specificity was 100% in the original study [27], but then dropped to 94% [89], 82% [44], and even 78% in the latter [42], which, however, included only nine controls.

In some cases, the percentage of apparently false-positive results in clinically diagnosed cohorts was related to a specific pathological condition, leaving different open interpretations. For example, in AD, LB-related co-pathology is a relatively frequent finding at post-mortem examination. Accordingly, the 7 out of 43 AD cases that were positive by RT-QuIC in the study by Rossi et al. [74] might have developed LBs as significant co-pathology, indicating a real positivity rather than a false positive readout.

Similarly, the evaluation of unexpected positive results in clinical “healthy” controls should take into account that Lewy pathology affects 5–8% of people over 60 in the absence of extrapyramidal signs [25, 28]. Since parkinsonian signs appear when about 30% of dopaminergic neurons of substantia nigra are lost [35], these subjects may represent prodromal-PD. The observed 100% specificity in the pre-senile control group of subjects examined by Rossi et al. [74] is consistent with this conclusion and seems to exclude spontaneous aggregation as the leading cause of false-positive results in their assay.

Concerning the diagnostic sensitivity, most studies demonstrated high values (above 90%) across the entire spectrum of syndromes associated with LB synuclein pathology [12, 27, 37, 52, 74, 89]. In a more in-depth analysis of the false-negative cases, clinical features did not show any specific association in any of the studies, suggesting that the negative response might be more likely due to the sample matrix composition rather than the underlying pathology.

The variable but generally very low sensitivity of α-syn RT-QuIC in MSA deserves a separate comment. Two studies to date analyzed a significant number of MSA cases. They reported a 6.1% [74] and 35% [89] overall sensitivity, indicating that α-syn RT-QuIC, in the currently proposed set-ups, consistently fails to reveal α-syn pathogenic species in the CSF of MSA patients. Thus, a negative rather than a positive result might be applied to support MSA’s clinical diagnosis. The striking difference in seeding activity in comparison to LBD specimens may rely on a different conformation of the pathogenic seed, referred to as strain, as suggested by recent in vitro and ex vivo studies [82].

Nonetheless, when compared to controls, CSF samples from MSA patients gave a higher number of positive reads than controls, suggesting a certain degree of cross-reactivity with the α-syn recombinant substrate. Thus, the use of novel substrates or the modification of other parameters in the RT-QuIC reaction might allow to increase the sensitivity for α-syn seeding activity in MSA cases and to distinguish them from controls and other synucleinopathies.

### CSF 4R-tau RT-QuIC: a potential diagnostic aid in the differential diagnosis of parkinsonism and cognitive decline?

Tau RT-QuIC represents the latest developed adaptation of the seeding conversion assays. Tau is a protein whose insoluble filaments are found in AD as well as in several other neurodegenerative diseases, collectively termed tauopathies. In vitro evidence [26, 80] indicated that tau possesses self-seeding properties resembling those of PrP. Therefore, following the success obtained in prion diseases and synucleinopathies, researchers also attempted to adapt the RT-QuIC for the detection of misfolded tau in the CSF.

However, the tau protein complexity arising from the presence of six isoforms, which differ one another for the inclusion of amino-terminal regions (0 N, 1 N, and 2 N, respectively) and either 3 or 4 microtubule-binding repeats (3R and 4R tau, respectively), challenged the development of tau RT-QuIC until very recently.

The first successful RT-QuIC assay for the detection of misfolded tau species in the CSF was put forward by Saijo and co-workers [78]. The assay was initially tuned to accurately detect Pick’s Disease (PiD), a tauopathy in which aggregates exclusively comprise the 3R tau isoform. However, the test only worked with brain tissue or post-mortem CSF as seeds, limiting its potential application in vivo.

Using similar RT-QuIC conditions, but employing different tau constructs, the same group adapted the RT-QuIC for the detection of tau misfolded species from mixed (3R/4R) tauopathies, such as AD and chronic traumatic encephalopathy patients, or 4R tauopathies, such as PSP and CBD [49, 79]. While the former assay, once again, only worked with a brain homogenate as seed, the 4R-specific construct also revealed promising results in the samples collected in vivo, although the accuracy was significantly lower in the CSF samples obtained in vivo compared to those collected post-mortem [49, 57, 78, 79]. In summary, tau RT-QuIC embodies enormous potential but needs to be refined and tested in a large cohort of patients before being proposed as a valuable diagnostic tool.

## Conclusions

After its original implementation 10 years ago [91], the RT-QuIC assay has gained significant ground among the available laboratory aids for the clinical diagnosis of neurodegenerative disorders. Although the assay does not distinguish between disease subtypes and its prognostic value is still far from well-established, RT-QuIC has become the most potent tool for the in vivo diagnosis of prion disease and has significantly impacted both clinical diagnostic criteria and clinical practice. Moreover, recent α-syn RT-QuIC set-ups provided a robust biomarker for LB-related disorders, supporting its introduction in research diagnostic criteria and clinical trials. Finally, promising preliminary results also involve misfolded tau and the primary 3R and 4R tauopathies.

Despite these significant advances, however, many challenges and unanswered questions remain. The recombinant substrate, which is critical for the RT-QuIC method’s accuracy, must be standardized and produced on a larger scale to be more readily available to laboratories worldwide. Most recent RT-QuIC protocols need validation in the inter-laboratory setting and the accuracy of the RT-QuIC using peripheral tissues such as the skin and nasal brushes further testing. In prion disease, there is also a need for improved RT-QuIC protocols to differentiate between sCJD subtypes and detect atypical prion diseases with higher sensitivity in vivo.

In synucleinopathies, future studies should establish the impact of RT-QuIC on clinical diagnostic criteria, determine the assay performance on other accessible specimens, and search for variations in the RT-QuIC set-up that might correctly detect α-syn seeding activity in MSA. Moreover, the application of RT-QuIC to genetically determined PD cases and other rare atypical forms of PD should help to establish the real spectrum of LB-related PD, and distinguishing PD associated with LB pathology from PD cases caused by primary neuronal degeneration in the substantia nigra without LB formation. Finally, protocols must be developed further to bring tau RT-QuIC to the level of diagnostic accuracy shown by the prion and α-syn assays. If successful, these and other advancements will likely make the RT-QuIC of critical importance for the early diagnosis and treatment of most of these devastating disorders.

## Data Availability

Not applicable.

## Abbreviations

RT-QuIC: Real-time quaking-induced conversion assay
PrP: Prion protein
PrP^C^: Cellular prion protein
PrP^Sc^: Scrapie prion protein
rPrPsen: Hamster recombinant prion protein
CJD: Creutzfeldt-Jakob disease
sCJD: Sporadic Creutzfeldt-Jakob disease
LBD: Lewy body disease
AD: Alzheimer’s disease
PK: Proteinase K
PMCA: Protein misfolding cyclic amplification
QuIC: Quaking-induced conversion
ThT: Thioflavin-T
α-syn: α-synuclein
GSS: Gerstmann-Sträussler-Scheinker disease
FI: Fatal insomnia
VPSPr: Variably protease-sensitive prionopathy
M: Methionine
V: Valine
RPDs: Rapidly progressive dementias
DW-MRI: Diffusion-weighted magnetic resonance imaging
IQ-CSF: Improved RT-QuIC CSF
SDS: Sodium dodecyl sulfate
PD: Parkinson’s disease
DLB: Dementia with Lewy bodies
MSA: Multiple system atrophy
LB: Lewy bodies
PAF: Pure autonomic failure
iRBD: Isolate REM (rapid eye movement) sleep behavior disorder
PSP: Progressive supranuclear palsy
CBD: Corticobasal degeneration
FTD: Frontotemporal dementia
PNS: Peripheral nervous system
MIBG: Metaiodobenzylguanidine
PSG: Polysomnography
EEG: Electroencephalography
MRI: Magnetic resonance imaging
FDG-PET: Fluorodeoxyglucose-positron emission tomography
LP: Lumbar puncture
LRRK2: Leucine-rich repeat 2 gene
PiD: Pick’s Disease
